# Enhancing oral bioavailability of quercetin using novel soluplus polymeric micelles

**DOI:** 10.1186/1556-276X-9-684

**Published:** 2014-12-18

**Authors:** Linghui Dian, Enjiang Yu, Xiaona Chen, Xinguo Wen, Zhengzan Zhang, Lingzhen Qin, Qingqing Wang, Ge Li, Chuanbin Wu

**Affiliations:** School of Pharmaceutical Sciences, Guangdong Medical College, Xincheng Road 1, Dongguan, 523808 Guangdong People’s Republic of China; School of Pharmaceutical Sciences, Sun Yat-Sen University, Waihuan Road 132, Guangzhou, Guangdong 510006 People’s Republic of China; R&D Center of Pharmaceutical Engineering, Sun Yat-sen University, Waihuan Road 132, Guangzhou, 510006 Guangdong People’s Republic of China

**Keywords:** Soluplus, Polymeric micelles, Oral bioavailability, Quercetin

## Abstract

To improve its poor aqueous solubility and stability, the potential chemotherapeutic drug quercetin was encapsulated in soluplus polymeric micelles by a modified film dispersion method. With the encapsulation efficiency over 90%, the quercetin-loaded polymeric micelles (Qu-PMs) with drug loading of 6.7% had a narrow size distribution around mean size of 79.00 ± 2.24 nm, suggesting the complete dispersibility of quercetin in water. X-ray diffraction (XRD) patterns illustrated that quercetin was in amorphous or molecular form within PMs. Fourier transform infrared spectroscopy (FTIR) indicated that quercetin formed intermolecular hydrogen bonding with carriers. An *in vitro* dialysis test showed the Qu-PMs possessed significant sustained-release property, and the formulation was stable for at least 6 months under accelerated conditions. The pharmacokinetic study in beagle dogs showed that absorption of quercetin after oral administration of Qu-PMs was improved significantly, with a half-life 2.19-fold longer and a relative oral bioavailability of 286% as compared to free quercetin. Therefore, these novel soluplus polymeric micelles can be applied to encapsulate various poorly water-soluble drugs towards a development of more applicable therapeutic formulations.

## Background

Oral administration is by far the easiest and most acceptable route of drug delivery, especially for the long-term medication of patients [1]. But about 40% of the approved active molecules have low solubility, resulting in poor oral bioavailability. Many efforts have been devoted to the development of oral sustained-release systems that can not only improve drug bioavailability leading to better efficacy and less administration frequencies but also decrease the fluctuation of plasma drug concentration to lower side effects [2]. In recent decades, emerging nanotechnology provides a novel platform to solve the solubility problem of drugs [3, 4]. Especially, polymeric micelles as a promising drug delivery system is a new research hotspot [5], most current studies concentrated on developing polymeric micelles for injection drug delivery [6]. Drug-loaded micelles in the systemic circulation characterizes long retention time and excellent tissue permeability and can gather in the diseased tissue to gain passive targeting [7, 8]. Furthermore, polymeric micelles with stable, biocompatible, and solubilizing properties have drawn considerable attention for oral administration.

Polymeric micelles with inner ‘core’ and outer ‘shell’ are formed by amphiphilic copolymers composed of hydrophilic and hydrophobic chains that can self-assemble in water above the critical micelle concentration (CMC) [9]. A polymeric micelle has the ability to encapsulate a hydrophobic drug into their cores and deliver the drug to the desired site at the concentration exceeding the intrinsic solubility of the drug. Moreover, the encapsulated drug can be not only protected from contact with the GI contents which likely induce degradation and metabolism but also conferred with the characteristics of sustained-release and direct uptake by cells. Many studies have proven that nanoparticles can transport across the intestinal membrane through paracellular or trancellular routes [10], while maintaining their integrity [11]. Therefore, the oral formulation based on nanosized polymeric micelles was expected to achieve the advantages of nanoparticles, such as enhanced permeability and retention (EPR) effects.

**Figure 1:**
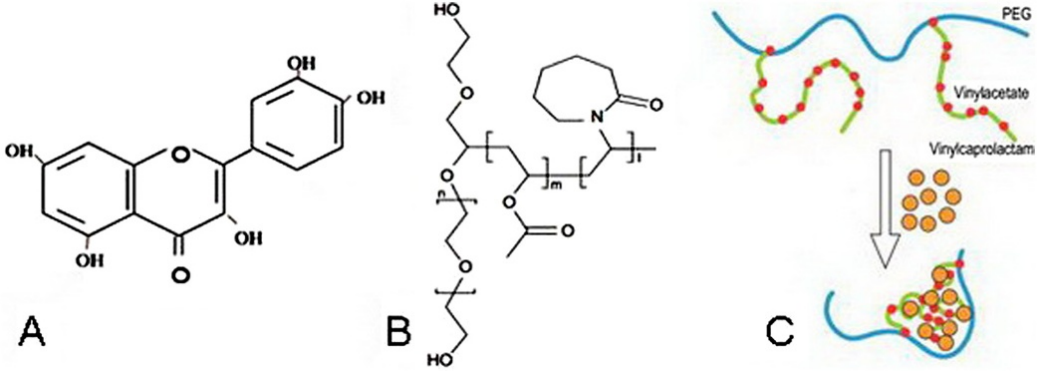
**The structure of quercetin (A), soluplus (B) and formation of PMs (C).**

Quercetin (3, 3′, 4′, 5, 7-pentahydroxy flavones, Figure 1A) is a flavonoid compound widely present in flower, leaf, and fruit of plants such as *Sophora japonica L.*, *Dendranthema morifolium* (*Ramat.*) *Tzvel*, and *Crataegus pinnatifida bunge*, with a variety of biological activity and high medical value [12]. Literature indicates that quercetin can inhibit the growth and proliferation of a variety of cancer cell lines (human ovarian cancer, breast cancer, lung cancer, human colon cancer, etc.) [13–16]. Quercetin can also lower the multidrug-resistance in cancer cells [17, 18] and enhance the antitumor effects of drugs [19, 20].

But clinical use of quercetin is limited due to its poor water solubility and instability in physiological media [21], leading to poor bioavailability [22]. Polymeric micelles are regarded as excellent candidates for anticancer drug delivery, and several anticancer drugs delivered by amphiphilic polymer micelles have already been preceded to clinical study or market [23, 24]. Recently, polymer micelles have been utilized for quercetin formulation too [25].

Soluplus, an amphiphilic polyvinyl caprolactam-polyvinyl acetate-polyethylene glycol graft copolymer (Figure 1B), was introduced by BASF. This graft copolymer has a polyethylene glycol (PEG) backbone as hydrophilic part and vinylcaprolactam/vinyl acetate side chains as lipophilic structure. So, micelles can be formed in aqueous solution above the CMC of 7.6 mg · L^-1^ (Figure 1C) [26].

The objective of this study was to develop a nanomicelle delivery system by using soluplus and poloxamer 407(F_127_), which could solubilize quercetin in aqueous media, reaching the clinically relevant concentration and delivering quercetin in a controlled manner. The previously reported preparation method was slightly modified to produce soluplus micelles with suitable size, charge, and stable properties. Powder X-ray diffraction (XRD) was employed to identify the physical state of quercetin in the polymeric micelles. Taking advantage of their permeation-enhancing effect, the soluplus nanomicelles were evaluated *in vivo* as potential vehicles, and the pharmacokinetic profile of orally administered quercetin encapsulated in micelles was investigated.

## Methods

### Materials

Soluplus^®^ was friendly supplied by BASF Auxiliary Chem. Co., Ltd. (Shanghai, China). Poloxamer 407 (PEO_98_POP_67_PEO_98_) was obtained from BASF (Ludwigshafen, Germany). Quercetin (Qu) was purchased from Shanxi Sciphar Biotech. Co. Ltd. (Shanxi, China). Methanol (high-performance liquid chromatography (HPLC) grade) was purchased from Fisher Scientific, Waltham, MA, USA. Milli-Q grade water purified through a Millipore system (ELGA LabWater, Sartorius, UK) was used throughout this study. All solvents were used without further purification.

### Animals

Beagle dogs were obtained from Experimental Animal Center of Sun Yat-sen University (Guangzhou, China). Beagle dogs were provided with standard food and water at will and were exposed to alternating 12-h periods of light and darkness. Relative humidity and temperature were maintained at 50% and 25°C, respectively. All care and handling of animals were performed with the approval of Institutional Authority for Laboratory Animal Care of Sun Yat-sen University.

### Preparation of quercetin-loaded soluplus polymeric micelles

Quercetin-loaded polymeric micelles (Qu-PMs) were formed by a modified film dispersion method using soluplus and F_127_ [27]. Briefly, soluplus (10 mg) and quercetin (7 mg) were dissolved together in organic solvent acetone, followed by evaporation under reduced pressure in a rotary evaporator at 35°C. The deionized water was then added into the polymer and drug solution, allowing the self-assembly of soluplus and quercetin to form quercetin-encapsulated polymeric micelles with core-shell structure (Figure 1C) at 650 rpm. Finally, the prepared Qu-PMs were lyophilized for future application.

### Determining the optimum concentration of F_127_

Then the optimum concentration of F_127_ required for preparing the desirable Qu-PMs was determined based on particle size and encapsulation efficiency.

### Determining the optimum stirring time

The optimum time of magnetic stirring for the preparation of Qu-PMs was determined on the basis of particle size and encapsulation efficiency.

### Optimization of drug loading

Qu-PMs were prepared using different theoretical Qu loading, i.e., 5%, 7%, and 9% of polymer on the basis of preliminary experiment, to determine the optimum percentage of Qu in soluplus matrix and its effects on particle size, polydispersity index (PDI), zeta potential, and encapsulation efficiency of Qu-PMs. The magnetic stirring time (2 h), stabilizer concentration (1% of F_127_), and aqueous ratio were kept constant.

### Characterization of Qu-PMs

#### Particle size and zeta potential measurements

The particle size and PDI of Qu-PMs were determined by using a Malvern Instruments Zetasizer Nano ZS90 (Malvern Instruments, Malvern, UK) on the basis of photon correlation spectroscopy. The dispersion of Qu-PMs was diluted in double distilled water and measured at 25°C for analysis. The particle size and PDI were obtained by cumulate analysis using the MALVERN software. The Zeta potential of Qu-PMs also measured by using a Malvern Instruments Zetasizer Nano ZS90 (Malvern Instruments, Malvern, UK). All experiments were repeated three times.

#### Transmission electron microscopy

The surface morphology of Qu-PMs was examined by using a transmission electron microscope (TEM; H66009IV, Hitachi, Chiyoda-ku, Japan). The dispersion of Qu-PMs were placed on a copper grid covered with nitrocellulose, negatively stained with phosphotungstic acid, and allowed to dry at room temperature.

#### X-ray diffraction

The X-ray diffraction patterns of pure Qu, void PMs, physical mixture of void PMs and Qu, and Qu-PMs were obtained by using an X-ray powder diffractometer (Bruker AXS, Madison, WI, USA) at a voltage of 40 kV and 25 mA with a scanned angle from 5*°* ≤ 2θ ≤ 50*°* at a scan rate of 0.9 · min^-1^.

#### Fourier transform infrared spectrometer

The Fourier transform infrared spectroscopy (FTIR) spectra of Qu, void PMs, physical mixture of void PMs and Qu, and Qu-PMs were recorded on a Nicolet 5700 FTIR spectroscopy (Thermo, Waltham, MA, USA) using a Smart OMNI-sampler accessory. The samples were put on KBr plates. The FTIR spectra were recorded at 1 cm^-1^ resolution, with the range of 400 to 4,000 cm^-1^.

#### Encapsulation efficiency (EE)

The content of Qu encapsulated in PMs was determined by membrane filter method. 0.5 mL of Qu-PMs was filtered through the 0.22-μm membrane, while non-encapsulated Qu was retained on the membrane. The filtrate which contained Qu-PMs was demulsificated with methanol and analyzed for entrapped Qu content by high-performance liquid chromatography (HPLC). All experiments were repeated three times.

#### In vitro release

*In vitro* release of Qu from Qu-PMs was undertaken by the dialysis bag method [28]. The dialysis bags (MWCO 14000, Millipore, Boston, MA, USA) were immersed in double-distilled water for 24 h prior to loading with 2 mL of Qu-PMs dispersion or quercetin solution (equivalent to 4 mg of Qu). The loaded bags were putted into a conical flask and soaked in 100 mL of 35% (*v*/*v*) ethanol, and the flask was placed in a water bath at 37°C ± 0.5°C and stirred rate of 100 rpm. The release medium (5 mL) was taken out at time intervals of 0.5, 1.0, 2.0, 4.0, 6.0, 8.0, 12.0, 24.0, 48.0, 72.0, 96.0, 120.0, 144.0, 168.0, 192.0, 216.0, 240 h and added with the same volume of fresh medium to adjust a sink condition [29, 30]. The content of Qu was determined by HPLC. Each test was carried out in triplicate.

The mechanism of Qu release from PMs was performed by fitting the release rate data into the following equations:
123

Here *y* represents the accumulative release percentage; *t* sampling time; *k*_1_, *k*_2_, and *k*_3_ release rate constants for Equations 1, 2, and 3, respectively; a_1_ ~ a_3_ are constants for Equations 1, 2, and 3.

#### Storage stability

To assess the stability of Qu-PMs, the freeze dried Qu-PMs were putted into 5-mL glass vials, sealed with plastic caps and placed in an accelerated stability chamber with temperature of 30°C ± 2°C and RH of 65% ± 5% for 6 months. The formulations were evaluated for changes in particle size, PDI, and entrapment efficiency, besides physical appearance and ease of reconstitution [31, 32].

#### In vivo pharmacokinetics study after oral administration

To compare the pharmacokinetics of Qu-PMs with those of pure Qu after oral administration, an animal experiment was in favor of the Ethical Committee of the Sun Yat-sen University (Guangzhou, China) and performed in accordance with the National Institute of Health and Nutrition Guidelines for the Care and Use of Laboratory Animals.

Six beagle dogs (1.2 to 2.0 years of age) weighing 12 to 14 kg were acclimatized in an environmentally controlled breeding room for 1 week, before fasting overnight before the experiments, but allowed to drink water only. These dogs were randomly distributed into two groups each made up of three dogs. Dogs in one group were given pure Qu dispersed in Milli Q water containing 0.3% (*v*/*v*) CMC-Na, while dogs in the other group were administered Qu-PMs dissolved in distilled water. All the formulations were administrated at an equivalent dose of Qu 16 mg · kg^-1^ by oral gavage. Blood samples of 3 mL were collected from the hind leg vein and placed into heparinized tubes at time intervals of 0.5, 1.0, 1.5, 2.0, 3.0, 4.0, 5.0, 6.0, 8.0, 10.0, 12.0, 24.0, 36.0, and 48.0 h after oral administration. The plasma was segregated and the samples were kept at -20°C until analysis.

Qu was assayed using an HPLC method. In sample analysis, a 100 μL plasma sample aliquot was blended with protein-precipitating methanol agent (200 μL) and 0.25% HCL (100 μL) were added and vortexed for 2 min, then heated at 50°C for 10 min. After centrifugation at 12,000 rpm for 10 min, the supernatant was analyzed by HPLC. Qu analysis was carried out by injecting an aliquot (100 μL) of sample into the HPLC column (an odyssil C_18_ column, 4.6 × 250 mm, 5 μm) with a precolumn (4.6 × 12.5 mm, 5 μm), using a mobile phase making up of methanol 0.2% phosphoric acid (60:40, *v*/*v*) at a flow rate of 1.0 mL · min^-1^. The detection wavelength was 375 nm [33].

Pharmacokinetic parameters were estimated with one compartmental model using the 3P87 software (published by the State Food and Drug Administration of China for pharmacokinetic study). The *C*_max_ (highest peak concentration), *T*_max_ (the time at which *C*_max_ reached), and the AUC_0-∞_ (the total area under the curve) were determined. The Mann-Whitney *U*-test was analyzed statistically. The results of the test were evaluated as mean values ± SD (standard deviation), and that of *p* < 0.05 analysis was significant statistically difference.

## Results and discussion

### Preparation of Qu-PMs

Different process variables including stabilizer concentration, magnetic stirring time, and theoretical drug loading were optimized for the preparation of Qu-PMs.

### Effect of stabilizer concentration

**Figure 2:**
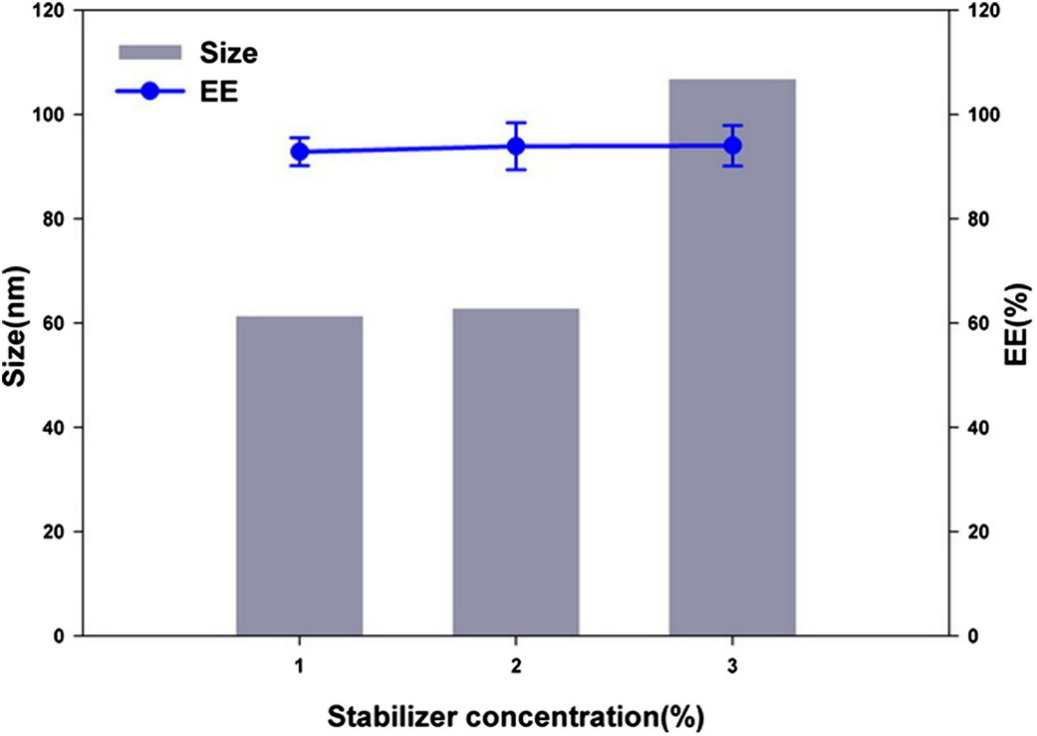
**Effects of stabilizer F**_**127**_ **concentration on size and EE of Qu-PMs (** ***n*** **= 3).**

The effects of F_127_ concentration on the size and encapsulation efficiency of Qu-PMs are shown in Figure 2. As the concentration of F_127_ increased (1% to 3%), the particle size of Qu-PMs increased, but the encapsulation efficiency remained almost constant at around 95%. The particle size with 3% F_127_ was larger than 100 nm which was unacceptable, and no significant difference was found in particle size and encapsulation efficiency with respect to 2% and 1% F_127_. So 1% F_127_ (*w*/*v*) was optimized for Qu-PMs preparation.

### Effect of stirring time

**Figure 3:**
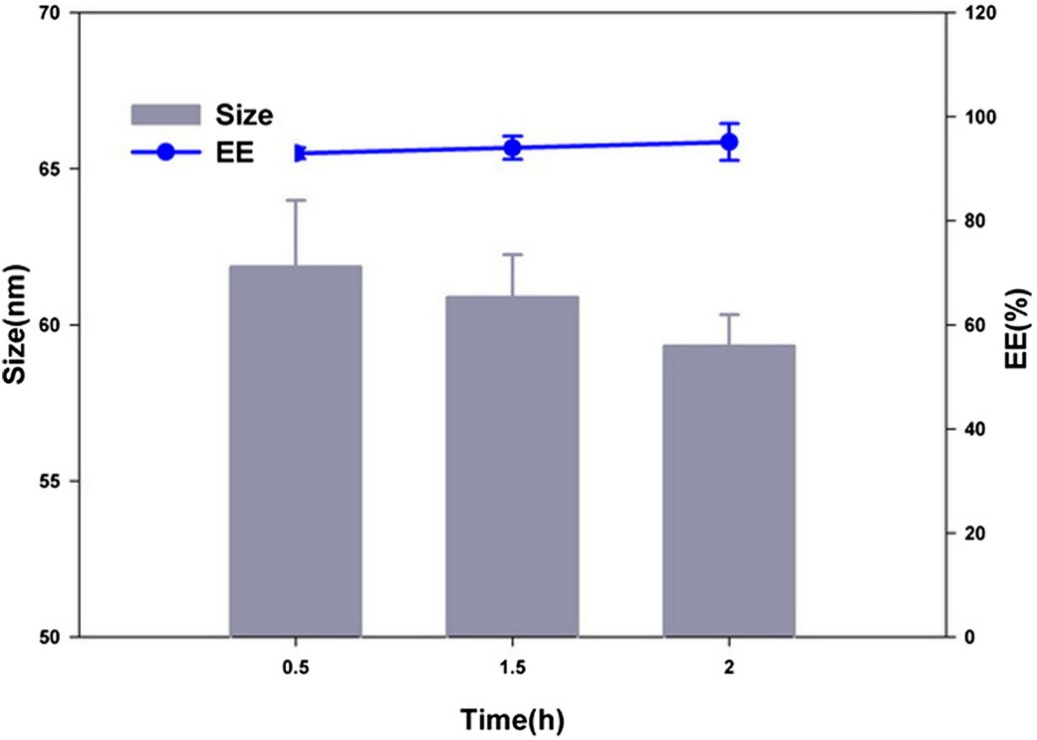
**Effect of magnetic stirring time on size and encapsulation efficiency (** ***n*** **= 3).**

Qu-PMs were prepared through magnetic stirring for particle size reduction. Figure 3 shows the decrease in particle size upon increasing the stirring time up to 2 h. Magnetic stirring of 2 h brought about the smaller particle size (59.32 ± 1.01 nm) and larger encapsulation efficiency (95.12% ± 3.54%) compared with shorter stirring time. So, magnetic stirring of 2 h was selected for further research.

### Optimum of drug loading

Qu-PMs were prepared with different theoretical drug loading, i.e., 5%, 7%, and 9% of polymer, to investigate the appropriate percentage of Qu in soluplus matrix. As shown in Table 1, initial drug loading affected the particle size and drug encapsulation efficiency of Qu-PMs significantly (*p* < 0.05). The particle size became big with the increase of drug loading, and the encapsulation efficiency also increased as drug loading increased from 5% to 7% but decreased significantly as drug loading further increased to 9%. Drug loading of 7% resulted in stable PMs with higher encapsulation efficiency (95.91% ± 4.05%), optimum particle size (79.00 ± 2.24 nm), and PDI (0.154 ± 0.044), as well as the most negative zeta potential (-17.10 ± 2.30). Taking this into consideration, the initial drug loading of 7% (*w*/*w*) was selected for Qu-PMs formulation.

**Table 1:** **Effects of initial drug loading on size, PDI, zeta potential, and EE (mean ± SD,** ***n*** **= 3)**

Drug loading (%)	Size (nm)	PDI	Zeta potential	EE (%)
5	59.97 ± 3.70	0.183 ± 0.023	-13.4 ± 0.20	93.24 ± 3.05
7	79.00 ± 2.24	0.154 ± 0.044	-17.10 ± 2.30	95.91 ± 4.05
9	111.2 ± 3.45	0.134 ± 0.082	-15.1 ± 1.60	75.06 ± 3.19

PDI, polydispersity index; EE, encapsulation efficiency; SD, standard deviation.

### Properties of Qu-PMs

#### Particle size and morphology of Qu-PMs

**Figure 4:**
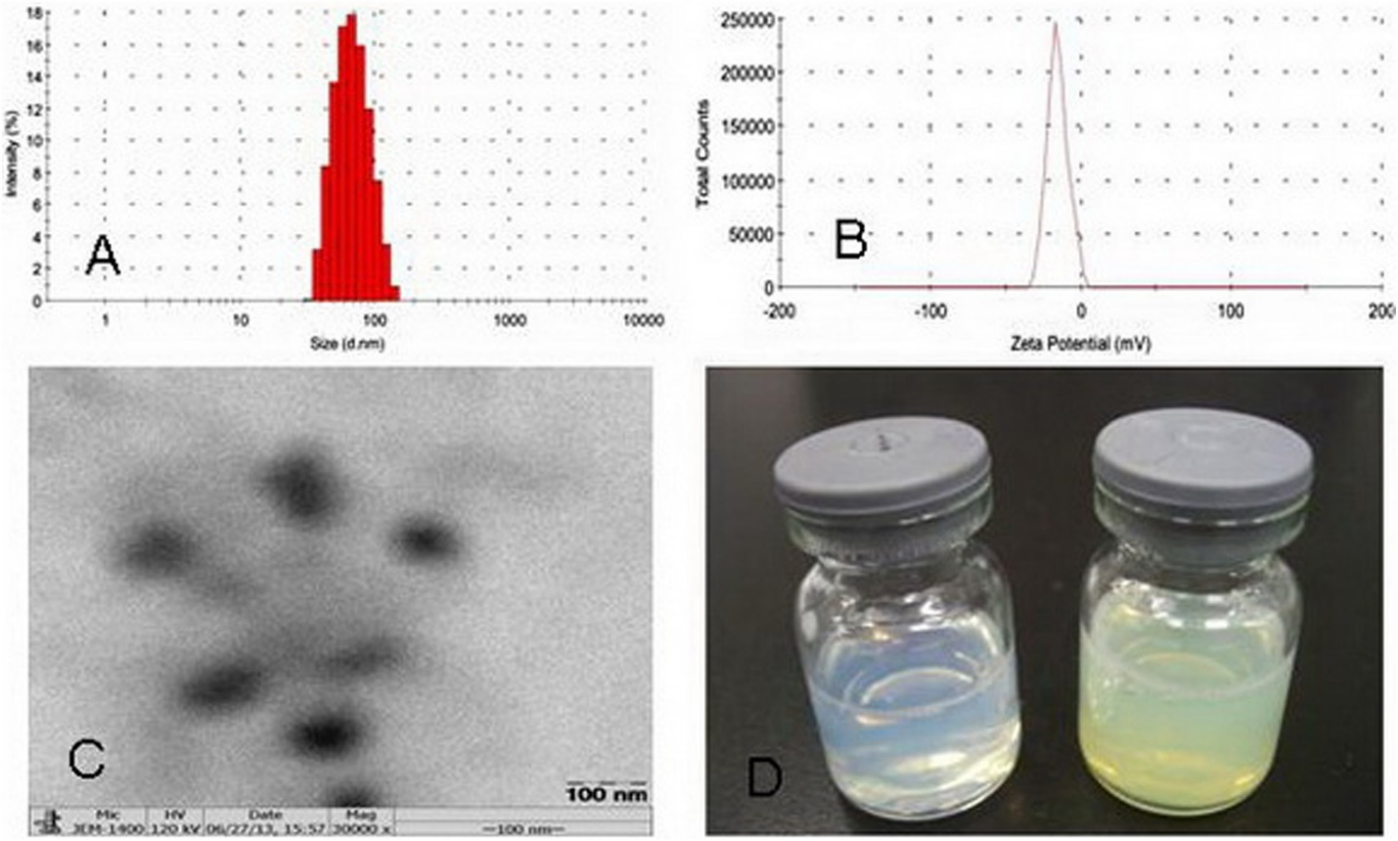
**Size (A), zeta potential (B), TEM (C), and colloidal solution (D) of Qu-PMs.**

Polymeric micelle size is a critical parameter for assessment of drug preparations. Studies reported that nanoparticles with sizes around or below 100 nm showed optimum cellular and nuclear uptake in epithelial and smooth muscle cells [34]. In the light of this, PMs with small size and high surface charge are expected favorable to intestinal uptake and extension of circulation half-life, as well as being evaded by the reticuloendothelial system (RES).The average particle size and the PDI of Qu-PMs were studied by dynamic light scattering. The representative size distribution of Qu-PMs (Figure 4A) clearly shows a narrow size distribution with the average particle diameter of 79.00 ± 2.24 nm and PDI of 0.154 ± 0.044. This conforms to the best particle size range for oral absorption. Zeta potential measurements possessed a negative surface charge of -17.10 ± 2.30 mV for Qu-PMs (Figure 4B), which certainly could increase the stability of Qu-PMs in dispersion. Mono-disperse and spherical Qu-PMs with a diameter of approximately 80 nm was examined by TEM (Figure 4C), which is consistent with the above results of dynamic light scattering. One major purpose of encapsulating Qu in PMs was to enable Qu to be completely dispersible in aqueous media, and this was confirmed the uniform solution of Qu-PMs with an opalescence (Figure 4D).

#### X-ray diffraction

**Figure 5:**
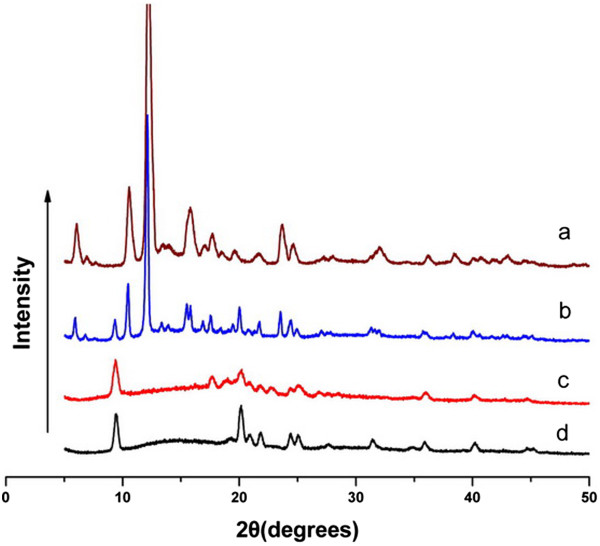
**XRD of Qu (a), physical mixture (b), Qu-PMs (c), and void PMs (d).**

The physical status of Qu encapsulated in PMs was compared with that of pure Qu by XRD analysis. The XRD patterns of pure Qu, void PMs, physical mixture of void PMs and Qu, and Qu-PMs are shown in Figure 5. In the figure, pure Qu exhibit a lot of distinct peaks that are traits of a crystalline structure [35]. The physical mixture of Qu and void PMs also present a number of distinct peaks, indicating that Qu is crystalline in the mixture. In the case of Qu-PMs, here were some characteristic peaks of Qu observed in 17°, which indicated that the drug was not completely amorphous in the PMs.

#### Fourier transform infrared spectrometer

**Figure 6:**
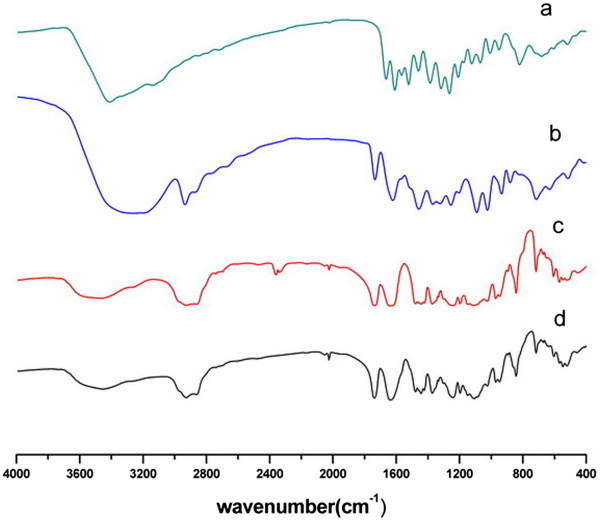
**FTIR of Qu (a), physical mixture (b), void PMs (c), and Qu-PMs (d).**

The molecular interactions within the solid matrix of the PMs were examined by FTIR method. On the basis of their structure, the possible interaction between quercetin and soluplus is hydrogen bonding, which may result in the shifting and peak broadening of the absorption bands at the interacting functional groups on the FTIR. The aromatic -OH group in quercetin might come into a hydrogen bond. FTIR of pure quercetin, void PMs, physical mixture of void PMs and quercetin, and Qu-PMs are presented in Figure 6. Pure quercetin showed a number of characteristic bands representing O-H stretching (3,700 to 3,300 cm^-1^), C = O absorption (1,670 cm^-1^), C-C stretching (1,612 cm^-1^), C-H bending (1,456, 1,383 and 866 cm^-1^), C-O stretching in the ring structure (1,272 cm^-1^), and C-O stretching (1,070 to 1,150 cm^-1^). The existence of these bands is consistent with the report of past studies [36]. Void PMs also show a number of bands, including OH stretch (3,500 to 3,250 cm^-1^), sp^3^CH stretching (2,932 cm^-1^), ester carbonyl stretching (1,742 cm^-1^), and C = O stretching for tertiary amide (1,641 cm^-1^). On the spectra of Qu-PMs, the position of carbonyl absorption peaks in Qu-PMs was shifted to 1737.93 cm^-1^ and 1636.80 cm^-1^, respectively. However, no similar peak shifting was observed in the physical mixture. These results illustrate that there might be some interactions between quercetin and soluplus in Qu-PMs.

#### Storage stability studies

The physicochemical properties of Qu-PMs containing 5% mannitol as a lyoprotectant were assessed after 6-month storage to establish their accelerated stability. Freeze dried Qu-PMs cakes were sealed in amber vials and stored in a stability chamber with temperature of 30 ± 0.5°C and relative humidity (RH) of 65% ± 5% for 6 months. The freeze-dried powder of Qu-PMs was stable without any shrinkage or collapse of the dried cake. The encapsulation efficiency, particle size, and PDI of the freeze dried Qu-PMs before and after storage were comparable (Table 2). Overall, these results suggested that the Qu-PMs had relatively good physical stability in the presence of mannitol under accelerated conditions and were able to protect majority (>90%) of the encapsulated bioactive component.

**Table 2:** **Characterization of freeze dried Qu-PMs with 5% mannitol after 6 months of storage at 30**°C **± 0.5**°C **and 65**% **± 5% RH (mean ± SD,** ***n*** **= 3)**

Parameters	Initial	Final
Size(nm)	63.76 ± 2.35	65.63 ± 3.71
PDI	0.151 ± 0.023	0.183 ± 0.056
EE (%)	92.06 ± 2.41	90.36 ± 3.84
Physical appearance	Intact cake	Intact cake
Ease of redispersion	By mere shaking	By mere shaking

PDI, polydispersity index; SD, standard deviation; EE, encapsulation efficiency.

#### In vitro release

**Figure 7:**
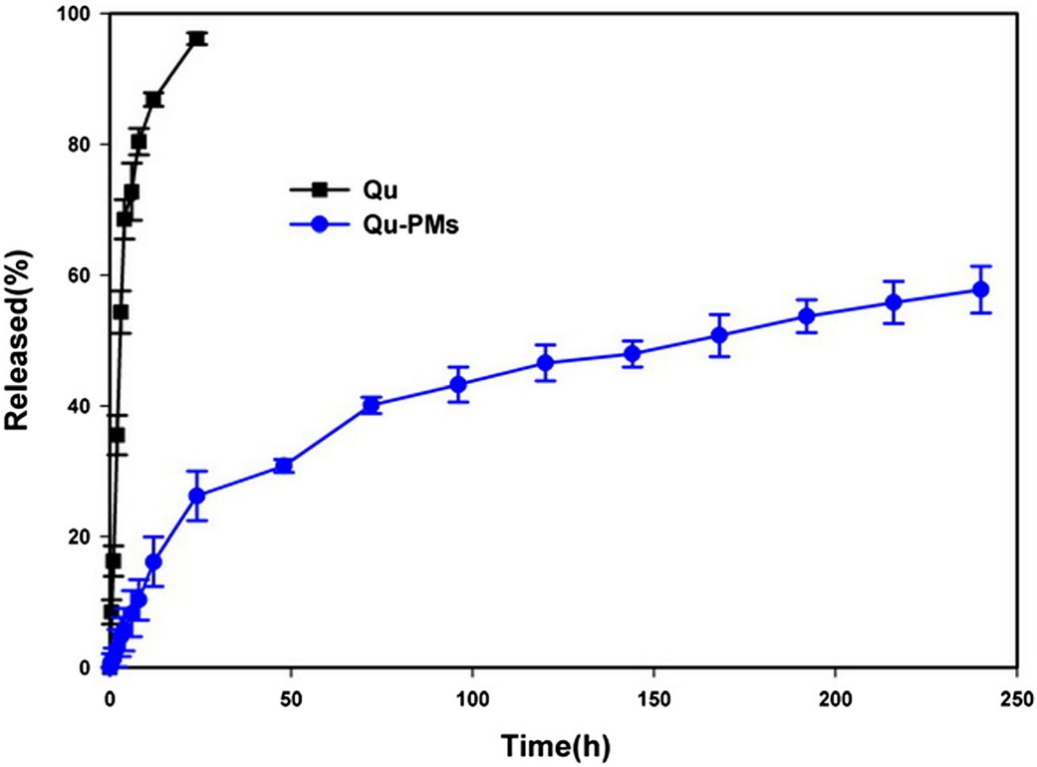
**Release of quercetin from propylene glycol solution and Qu-PMs suspension.**

As quercetin is insoluble in water, it simulated gastric fluid and intestinal fluid at room temperature (7.7, 5.4, and 28.87 μg · mL^-1^, respectively) [37]. Ethanol (35% (*v*/*v*)) was used as a receptor medium to obtain a sink condition in the dynamic dialysis study [33]. Drug release from quercetin contained propylene glycol solution and Qu-PMs suspension through the dialysis membrane at 37°C was shown in Figure 7. The pure quercetin from the solution showed about 96.13% release for a period of 24 h, during which no more than 26.22% of quercetin was released from Qu-PMs. Qu-PMs suspension exhibited sustained-release property and the accumulated release at 240 h is only 57.78%. The sustained release may be attributed to the diffusion of quercetin entrapped within the core of PMs.

In addition, the release data of Qu-PMs were fitted into different release mechanism models (Table 3). A linear relationship was established between the Qu release rate and the square root of time (*R*^*2*^ > 0.98), suggesting the release kinetics can be explicated by Higuchi’s equation. Namely, Qu is released from PMs by diffusion [38]. The reason for sustained drug release may be the formation of hydrogen bonds between drug and carrier molecules, which hinders the drug release. For the same reason, quercetin was entrapped in the polymeric micelle hydrophobic cores [39] _,_ and the nanoencapsulation of quercetin in PMs may improve the bioavailability of this molecule significantly.

**Table 3:** **Fitting of Qu release data from Qu-PMs into various mechanism models**

Model	Equation	***R*** ^2^
Zero-order	*y* = 0.246 *t* + 8.291	0.8766
First-order	ln(*1* - *y*) = 0.004 *t* - 16.129	0.9311
Higuchi	*y* = 4.018 *t1*/2 - 0.081	0.9894

*y*, accumulative release percentage; *t*, sampling time; *R*, correlation coefficient.

#### In vivo pharmacokinetics

**Figure 8:**
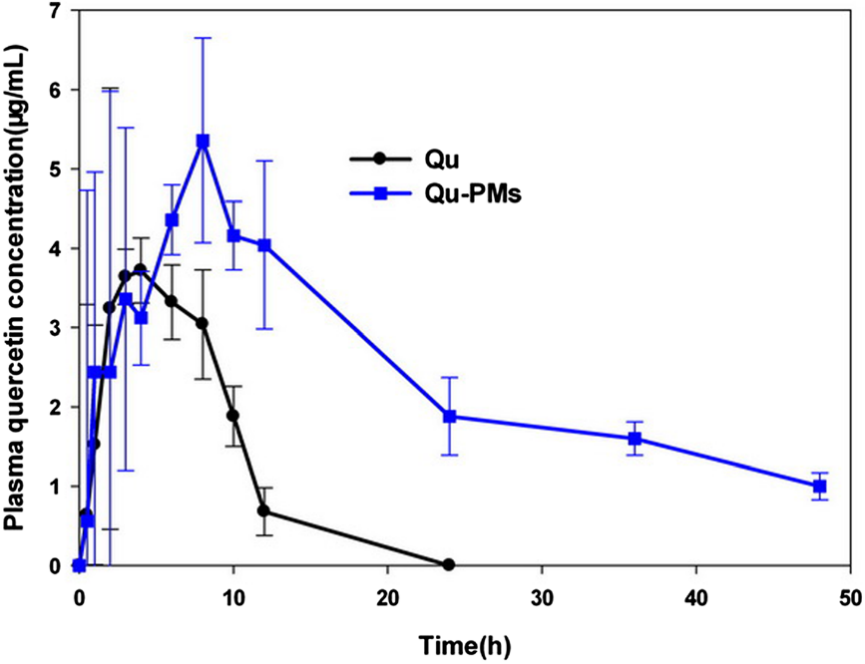
**Mean quercetin plasma concentration.**

It has been reported that quercetin is found in plasma as conjugates of glucuronic acid and sulfate groups [40]. Quercetin is released from the binding complex by acid hydrolysis method, and total content of quercetin in plasma was determined by HPLC [41]. Calibration samples were obtained by joining proper volumes of denominator Qu solution in methanol into blank plasma, gaining a calibration curve over the detected level range of 0.10 to 8.00 μg · mL^-1^ (*R*^2^ > 0.99). The results of the method validation ascertained by assessing the precision, accuracy, recovery, and limit of quantification and proved that the method was reliable. The bioavailability of Qu-PMs was looked into by investigating beagle dogs and comparing with that of pure Qu. After oral administration of a single dose equivalent to 16 mg · kg^-1^ of pure Qu or Qu-PMs, the mean quercetin concentrations in dog serum at different time intervals are plotted in Figure 8 and the calculated key pharmacokinetic parameters are summarized in Table 4. The plasma level of quercetin was detected only up to 24 h after administration of free drug, with the *C*_max_ of 5.24 μg · mL^-1^. The drugs released from PMs were still detected in plasma 48 h after oral administration, with the *C*_max_ of 7.56 μg · mL^-1^. As shown in Figure 8, after oral administration, Qu-PMs were absorbed much slower than pure quercetin with *T*_max_ of 7.02 ± 2.02 h and 5.31 ± 1.08 h (*p* < 0.05), respectively.

**Table 4:** **Pharmacokinetic parameters of quercetin in serum after oral administration (mean ± SD,** ***n*** **= 3)**

Parameter	Pure Qu	Qu-PMs
*C*_max_ (μg/mL)	5.24 ± 1.32	7.56 ± 3.28
*T*_max_ (h)	5.31 ± 1.08	7.02 ± 2.02
AUC_0~∞_(μg/h/mL)	37.68 ± 16.8	107.84 ± 54.4
*T*_1/2_ (h)	4.94 ± 2.03	10.81 ± 3.7
MRT (h)	7.18 ± 2.25	27.09 ± 7.8
*F* (%)		286 ± 3.23

AUC_(0→∞)_, area under the plasma concentration-time curve calculated by the linear trapezoidal rule from time 0 to infinity; SD, standard deviation; *T*_1/2_, elimination half-life; *C*_max_, peak plasma concentration; *T*_max_, time to reach peak plasma concentration.

What is more, the half-life (*T*_1/2_) of Qu-PMs was 2.19-fold longer than that of pure quercetin (Table 4), indicating a maximum residence time (MRT) of quercetin in the systemic circulation remarkably extended for Qu-PMs after oral administration. As the AUC_0*–∞*_ value of Qu-PMs was significantly larger than that of pure quercetin, the relative oral bioavailability of Qu-PMs calculated from AUC_0*–∞*_ values was about 286% (*p* < 0.05) comparing with pure quercetin. These results implied an enhanced bioavailability of quercetin was achieved through incorporation of drug into PMs.

The main purpose of this study was to prepare an aqueous formulation which could improve the oral bioavailability of the hydrophobic quercetin. In this regard, a nanocarrier system based on soluplus PMs was developed in this study and as anticipated, the pharmacokinetic results indicated the bioavailability of the delivered quercetin was enhanced. When administered via oral route, PMs may be absorbed through specialized M-cells of the Peyer’s patches in the small intestine [1]. Though the PMs showed the potential to enhance the oral bioavailability of poorly water-soluble drugs, the underlying mechanisms of enhancement, however, are still unclear and provoke future research interests.

## Conclusions

Quercetin was loaded into nanosized polymeric micelles based on amphiphilic polymers soluplus using a modified film dispersion method. The stable Qu-PMs showed sustained release of entrapped quercetin for up to 10 days *in vitro*, and more importantly, a sustained plasma level and enhanced systemic bioavailability of quercetin *in vivo*. Thus, the Qu-PMs provide a promising carrier candidate with efficient delivery of quercetin for therapeutic treatment in near future. Moreover, this study explores an interesting alternative approach for design and fabrication of novel polymeric micelles as delivery systems for bioactive compounds.
